# Increased protein aggregation in Zucker Diabetic Fatty rat brain: identification of key mechanistic targets and the therapeutic application of hydrogen sulfide

**DOI:** 10.1186/1471-2121-15-1

**Published:** 2014-01-06

**Authors:** Fatemeh Talaei, Veroniek M Van Praag, Mahdi H Shishavan, Sjoerd W Landheer, Henk Buikema, Robert H Henning

**Affiliations:** 1 Department of Clinical Pharmacy and Pharmacology, University Medical Center Groningen, University of Groningen, PO Box 196, 9700 AD Groningen, The Netherlands

**Keywords:** Autophagy, ZDF rats, Protein aggregates, Cystathionine beta synthase, Carboxymethyllysine, NaHS, Fibronectin, Reactive oxygen species, mTOR

## Abstract

**Background:**

Diabetes and particularly high blood glucose levels are implicated in neurodegeneration. One of the hallmarks of neurodegeneration is protein aggregation. We investigated the presence of protein aggregation in the frontal brain of Zucker diabetic fatty (ZDF) rats, an animal model for diabetes. Further, the effect of NaHS in suppressing protein aggregation in cultured brain slices from ZDF was assessed.

**Results:**

The levels of protein synthesis, protein/gene expression, autophagy and anti-oxidant defense were evaluated in ZDF and control (Lean) brains.

Compared to Lean, ZDF brains displayed a significant increase in protein aggregates, p-tau, fibronectin expression and protein glycosylation. Increased phosphorylation of mTOR and S6 ribosomal protein in ZDF indicated higher protein synthesis, while the increase in ubiquitinated proteins and LC3-I in ZDF brains accompanied by lower LC3-II expression and LC3-II/LC3-I levels indicated the blockage of proteolytic pathways. CBS (cystathionine beta synthase) protein and mRNA expression and thiol group levels in ZDF brains were lower compared to Lean. ZDF brains show a higher level of reactive oxygen species. *In vitro* NaHS treatment normalized proteostasis while counteracting oxidative stress.

**Conclusion:**

Our data demonstrate increased protein synthesis and aggregation in the diabetic ZDF rat brain, which was reversible by NaHS treatment.

This is the first report on the potential use of NaHS as a novel strategy against protein aggregation in diabetic brain.

## Background

Impairment of cognitive function is a feature that has been observed in diabetes, especially in type II diabetic patients [1,2], although the relationship remains disputed. Increased glycated hemoglobin 1 (HbA_1c_), indicating increased average blood glucose levels over a longer period, is related to lower cognitive function in individuals with type 2 diabetes and the decline in glycemic control is related to lower scores on cognitive function tests [3]. In turn, this is in accordance with the finding of advanced glycation end-products (AGEs) in dementia and diabetic microvascular disease, in which decreased cerebral glucose metabolism is observed [4]. Indeed, low rates of glucose metabolism in brain cells lead to the accumulation of glycoproteins associated with subsequent formation of cytotoxic aggregates in brain [5]. Animal experiments also suggest a relationship between diabetes and cognitive impairment, as observed in type 2 diabetic models of genetically obese Zucker rat or db/db mouse [6] and in type 1 diabetic models of streptozotocin-induced diabetes mellitus in rat [7] and mouse [8]. As neurodegenerative diseases are often characterized by defects in brain protein maintenance [9], we employed an inbred model for type 2 diabetes the fa/fa Zucker diabetic fatty (ZDF) rat to study the effects of high glucose concentration on protein expression in brain. Moreover, there is still a lack of strategies to inhibit diabetes induced toxic protein aggregate formation in tissues which could be to some degree answered by our research.

Protein aggregate formation affects cellular function and has been implicated in various aging related disorders [10] and complications [11]. Protein aggregation is a common feature of neurodegenerative diseases leading to neurofibrillary tangle formation with cognitive deficits [12], including Alzheimer’s, Parkinson’s and Huntington’s disease, amyotrophic lateral sclerosis, frontotemporal dementia, ataxias, and human prion diseases. In many neurodegenerative complications such as Alzheimer’s, these tangles contain PHF (paired helical filaments) which are highly insoluble structures, composed of a highly phosphorylated form of the microtubule-associated protein tau. Although the characterization of brain protein aggregation is still insufficiently reported in type 2 diabetes, the close similarity in protein aggregate formation between Diabetes and Alzheimer’s may indeed indicate that the two conditions share key mechanistic targets [13]. It has been suggested that mTOR (mammalian target of rapamycin), which is an established component in proteostasis known to be implicated in different neurodegenerative complications [14], may represent an important link between nutrient excess with obesity and insulin resistance and their possible complications [15]. Although mTOR is an important regulator of neuronal development and function, including tau expression [16], to date only few studies have addressed a possible implication of mTOR in diabetes in relation to protein aggregate formation in brain tissue. Considering the induction of mTOR by high glucose levels [17], the mTOR pathway is likely implicated in the induction of brain protein aggregation in diabetes [18]. The presence of oxidative stress due to reactive oxygen species (ROS), and increased protein glycosylation leading to protein-protein crosslinking or aggregation [19] might be additional factors involved.

Different mechanisms deal with misfolded proteins to prevent the accumulation of aggregates. One such mechanism is autophagy, which removes ubiquitinated misfolded protein aggregates. In the light of possible mTOR activation (see above) [20], autophagy may be inhibited by phosphorylation of ribosomal protein S6, a main downstream effector of mTOR [21]. In addition, degradation by the ubiquitin-proteasome system seems involved in the clearance of misfolded proteins, especially oxidative stress induced ubiquitinated (UB) proteins that are resistant to breakdown via autophagy and ubiquitin routes [22]. Although acute oxidative stress induces autophagy [23], it is still conceivable that in diabetes the chronically increased levels of glucose may lead to imbalances in the antioxidant capacity within the cell. In turn, this would result in oxidative stress-mediated injury [24] which may impair autophagic clearance routes as observed in cells cultured at high glucose concentrations [25], thus further contributing to the formation of highly insoluble aggregates.

Another defense mechanism against protein aggregate formation constitutes of brain antioxidants such as FABP, thiol groups or H_2_S which would restrict oxidative damage by high glucose concentrations. It is known that pyridoxamine, an antioxidant and allosteric activator of the H_2_S producing enzyme cystathionine beta synthase (CBS) inhibits AGE formation [26] and protects cells from oxidative damage [27]. We have previously shown that H_2_S, as NaHS, modulates mTOR pathway in aging Werner syndrome skin fibroblasts and inhibits protein aggregation and aging phenotype while diminishing oxidative stress [28]. Further H_2_S has been also shown to induce protective autophagy in colon epithelial cells [29]. Nevertheless, the effect of H_2_S on inhibition or reversal of protein aggregation has yet not been studied in diabetic brain.

The first aim of this study was to identify diabetes induced protein aggregation in brain tissue obtained from an animal model of diabetes mellitus type 2. As frontal brain is implicated in cognitive behavior [30], we established the nature of protein aggregates in this brain region in 17 weeks old Zucker diabetic fatty rats (ZDF) compared to age matched Lean controls, and explored proteostasis routes. As we found changes in brain proteostasis to coincide with lower levels of brain thiol groups and diminished expression of CBS in ZDF, we sought as a second aim to investigate a potential beneficial effect of H_2_S. However, others have previously demonstrated that administration of H_2_S (as NaHS) influences glycemic control *in vivo* thus changing blood glucose and HbA_1c_ levels [31,32]. Therefore, we examined the effect of NaHS treatment on proteostasis in cultured brain tissue slices to assess the direct effects of H_2_S on brain, while excluding confounding effects of the modulation of glycemic control.

## Results

### HbA_1c_ and glucose concentrations

Development of diabetes in ZDF was monitored by measurement of HbA_1c_, glucose and body weight (Table 1). Body weight of ZDF animals was significantly increased compared to age matched Lean controls at all time points examined. Moreover, the increase in body weight of ZDF animals between week 7 and 17 (100 ± 4% (p < 0.05)) was 27% higher compared to the increase observed in Lean animals between week 7 and 17 (73 ± 2% (p < 0.05)). The impairment of glycemic control in ZDF animals was evidenced by both significantly higher blood glucose and HbA_1c_ levels compared to Lean at all time points examined. At week 17 in ZDF the percent difference to Lean amounted to 25 ±0.5% and 30 ±0.4% (p < 0.05) for blood glucose and HbA_1c_ respectively. The increase in blood glucose of ZDF animals between week 7 and 17 (19 ± 2% (p < 0.05)) was 13% higher compared to the increase observed in Lean animals between week 7 and 17 (6 ± 0.5% (p < 0.05)). In addition, the increase in HbA_1c_ level of ZDF animals between week 7 and 17 (50 ± 2% (p < 0.05)) was 33% higher compared to the increase observed in Lean animals between week 7 and 17 (17 ± 3% (p < 0.05)). Finally, the glucose concentration in the brain homogenates of the ZDF group (2.9 ± 0.7 mmol/kg) was found to be significantly higher than in the Lean group (1.3 ± 0.1 mmol/kg, p < 0.05). Together, these data demonstrate significantly impaired glycemic control in ZDF, yet without the full blown development of overt diabetes type 2.

**Table 1:** Development of diabetes in ZDF (zucker fatty rat) was monitored by measurement of HbA1c, glucose and body weight

	**Wk 7**	**Wk 9**	**Wk 11**	**Wk 13**	**Wk 15**	**Wk 17**
Glucose (mmol/l)						
Lean	7.8±0.4	8.9±0.1	8.3±0.2	8.3±0.2	8.5±0.3	8.3±0.2
ZDF	9.2±1.4*	9.9±0.6*	9.2±0.4*	9.6±0.4*	10.6±1.0*	11.0±1.0*
HbA1c (%)						
Lean	2.9±0.01				3.4±0.02	3.4±0.05
ZDF	3.3±0.04*				4.8±0.13*	5.0±0.24*
Body weight (g)						
Lean	211±5	247±4	280±3	317±5	314±6	365±5
ZDF	252±7*	332±9*	395±7*	449±5*	486±7*	504±9*

Body weight, HbA1c levels, and blood glucose levels are significantly increased in ZDF compared to Lean rats as measured in week 7 to week 17. * < 0.05 = different from controls (Lean) at the same stage.

### Protein aggregates, tau and fibronectin expression

**Figure 1:**
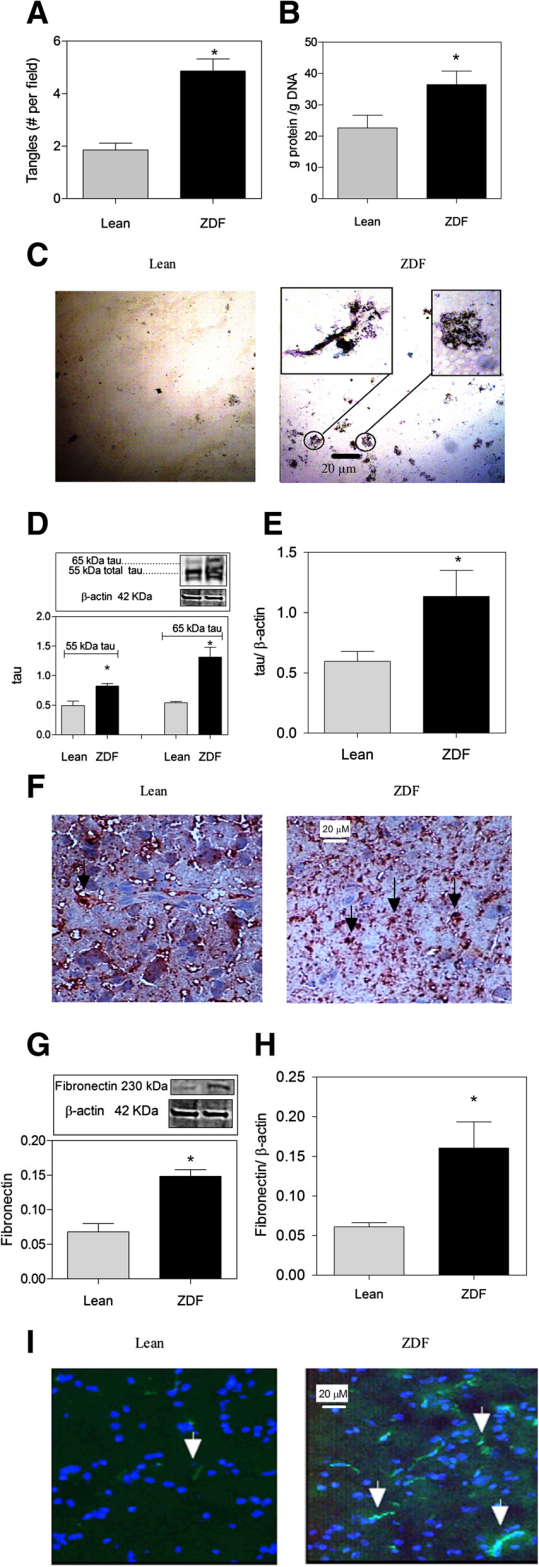
**Excess protein aggregation in the frontal brain of ZDF (zucker diabetic fatty rat) compared to Lean. (A)** ZDF brain shows doubling of the expression of protein aggregates (tangles). **(B)** Higher protein synthesis levels in ZDF brains indicated by higher protein/DNA ratio. **(C)** ZDF brain shows increased number of neurofibrillary tangles. Silver staining depicts tangles in dark brown/black elements with a diameter of 10–20 μm as magnified in top inset. **(D)** ZDF displays increased levels of tau proteins as found by Western blotting. **(E)** Real-time PCR analysis of the mRNA expression of total tau relative to β-actin. **(F)** ZDF displays increased levels of tau protein isomers and tau protein cleavage as found by immunohistochemistry (indicated by →). **(G)** ZDF displays increased levels of fibronectin as found by Western blotting. **(H)** Real-time PCR analysis of the mRNA expression of fibronectin relative to β-actin. **(I)** Immunohistochemistry analysis shows higher fibronectin expression in ZDF rats in comparison with Lean (indicated by →). Magnification 40×, Data are Means ± SEM (n *≥* 5 per group), * <0.0001 (difference to control gray bars in each group); unpaired t-test. Western blot expression is normalized to β-actin; lanes of western blot insets are in the same order as in the X-axis.

The silver stain showed a doubling of the number of protein aggregates present throughout the frontal brain in ZDF (Figure 1A). The aggregates were seen as black/brown entities with a tangled conformation (Figure 1C). To further substantiate increased protein synthesis in ZDF brains, the ratio of protein-to-DNA was measured. This ratio was significantly increased in ZDF brains showing higher protein synthesis levels in ZDF brains (Figure 1B). The expression of tau and fibronectin were investigated by Western blot analysis and qPCR analysis. Tau protein levels were increased in ZDF brains compared to Lean (Figure 1D). Especially tau protein isoforms with molecular weights around 55 and 65 kDa [33] were increased in frontal sections of ZDF compared to Lean (Figure 1D). In immunohistochemistry analysis, tau protein was seen as black/brown entities with an intact pattern in Lean brain but a dispersed pattern in ZDF brain (Figure 1F), which may suggest the cleavage of tau protein in ZDF. Total tau mRNA levels also showed a two times increase in ZDF brains (Figure 1E). Fibronectin protein expression was investigated because of its higher level of expression in diabetes [34] and also its presence in neurodegenerative diseases [35]. We observed that fibronectin protein in frontal brain of ZDF was about 3 times higher compared to Lean (Figure 1G). This was also confirmed by immunohistochemistry analysis of brain tissue (Figure 1I). Fibronectin mRNA levels were also found to be 3 times higher in ZDF brains compared to Lean (Figure 1H).

### Proteostasis components

**Figure 2:**
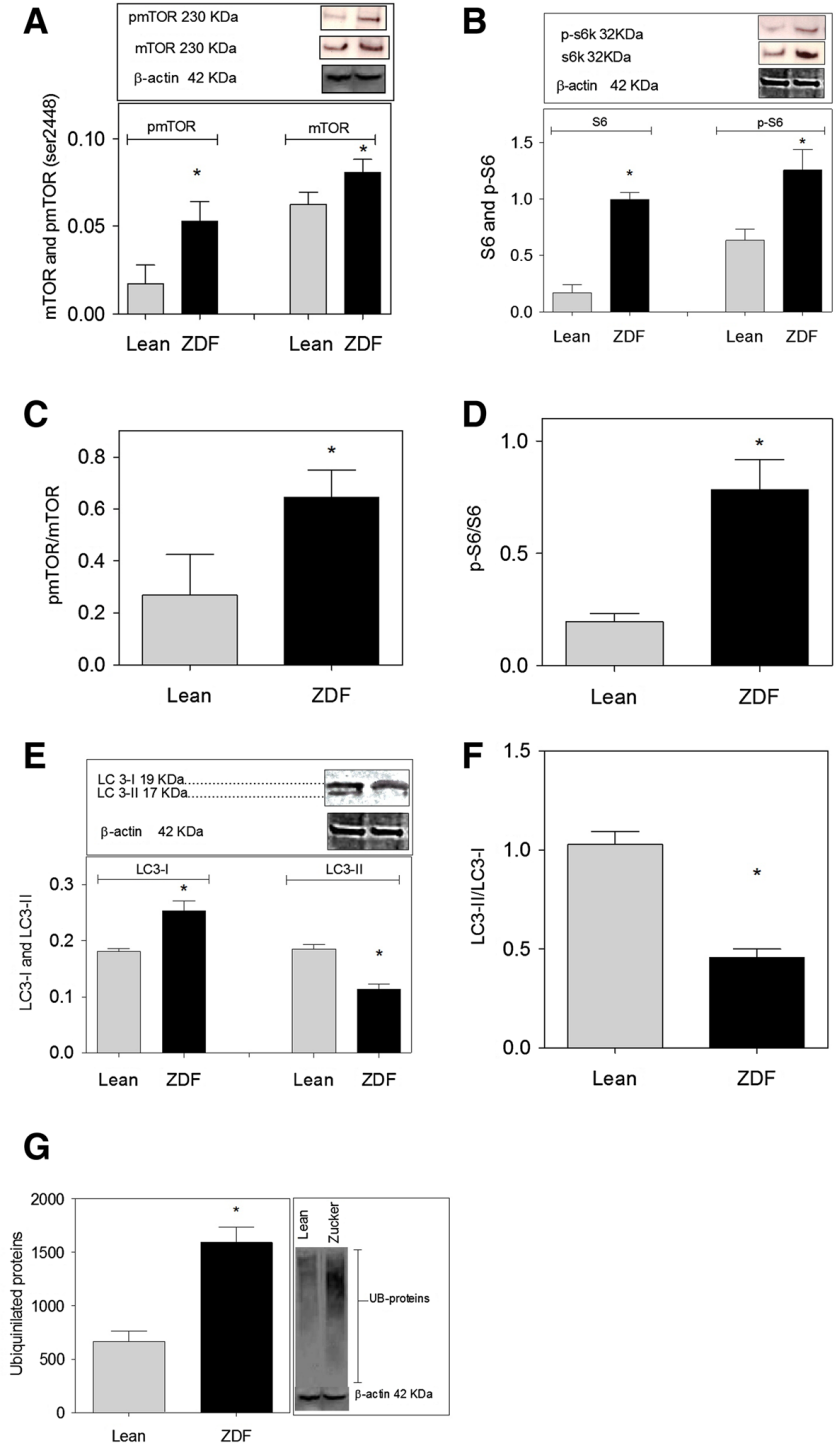
**mTOR pathway activity, autophagy and protein ubiquitination in brain of ZDF (zucker diabetic fatty rat). (A)** Increased expression of mTOR and its phosphorylated form in ZDF detected by an antibody raised against phosphorylation on serine 2448. **(B)** Increased S6 ribosomal protein (S6) and its phosphorylated form (p-S6) in ZDF. **(C)** Increased mTOR activity in ZDF. **(D)** Increased S6 protein activity in ZDF. **(E)** Increased expression of LC3-I and the decrease in LC3-II levels in ZDF. **(F)** Autophagy levels are lower in ZDF compared to Lean as shown by LC3-II/LC3-I ratio. **(G)** Increased levels of mono- and polyubiquitinated proteins in ZDF. Insets: typical examples of Western blot data showing protein of interest (upper part) and β-actin (lower part). Data are Means ± SEM (n *≥* 5 per group), * <0.0001 (difference to control gray bars in each group); unpaired t-test. Western blot expression is normalized to β-actin; lanes of western blot insets are in the same order as in the X-axis.

Increased expression of proteins such as tau and fibronectin in the face of aggregate formation may denote derailment of proteostasis in neurons. Therefore, we examined activation of the mTOR pathway as a major route controlling protein synthesis, as well as protein degradation by autophagy and ubiquitylation. ZDF brain demonstrated an increased expression of mTOR and phosphorylation of mTOR (p-mTOR ^serine 2448^) in ZDF (Figure 2A). Next, we examined ribosomal protein S6 (S6), being the immediate downstream target of the mTORC1 pathway conveying increased protein synthesis. In brain of ZDF, expression of both S6 and phosphorylated S6 (p-S6) was markedly elevated compared to Lean (Figure 2B). The ratios of the phosphorylated forms of mTOR and S6 ribosomal protein over the total expression of these proteins in brain was increased for both mTOR (Figure 2C) and S6 (Figure 2D) in ZDF brains, disclosing the activation of the mTOR pathway. To examine autophagy, Western blotting for LC3 (Microtubule-associated protein 1 light chain 3) was performed. ZDF rats showed an increased expression of LC3-I, with lower expression of LC3-II compared to Lean (Figure 2E). The ratio of LC3-II-to-LC3-I in ZDF brains is halved compared to Lean brain showing lower autophagy levels in ZDF brains (Figure 2F). Also, ZDF brains showed increased levels of mono- and polyubiquitinated proteins (Figure 2G). Collectively, these data substantiate the impairment of proteostasis in ZDF brain consisting of increased protein synthesis and degradation via the proteosomal route accompanied by lower autophagy levels.

### Glycosylation and reactive oxygen species damage

**Figure 3:**
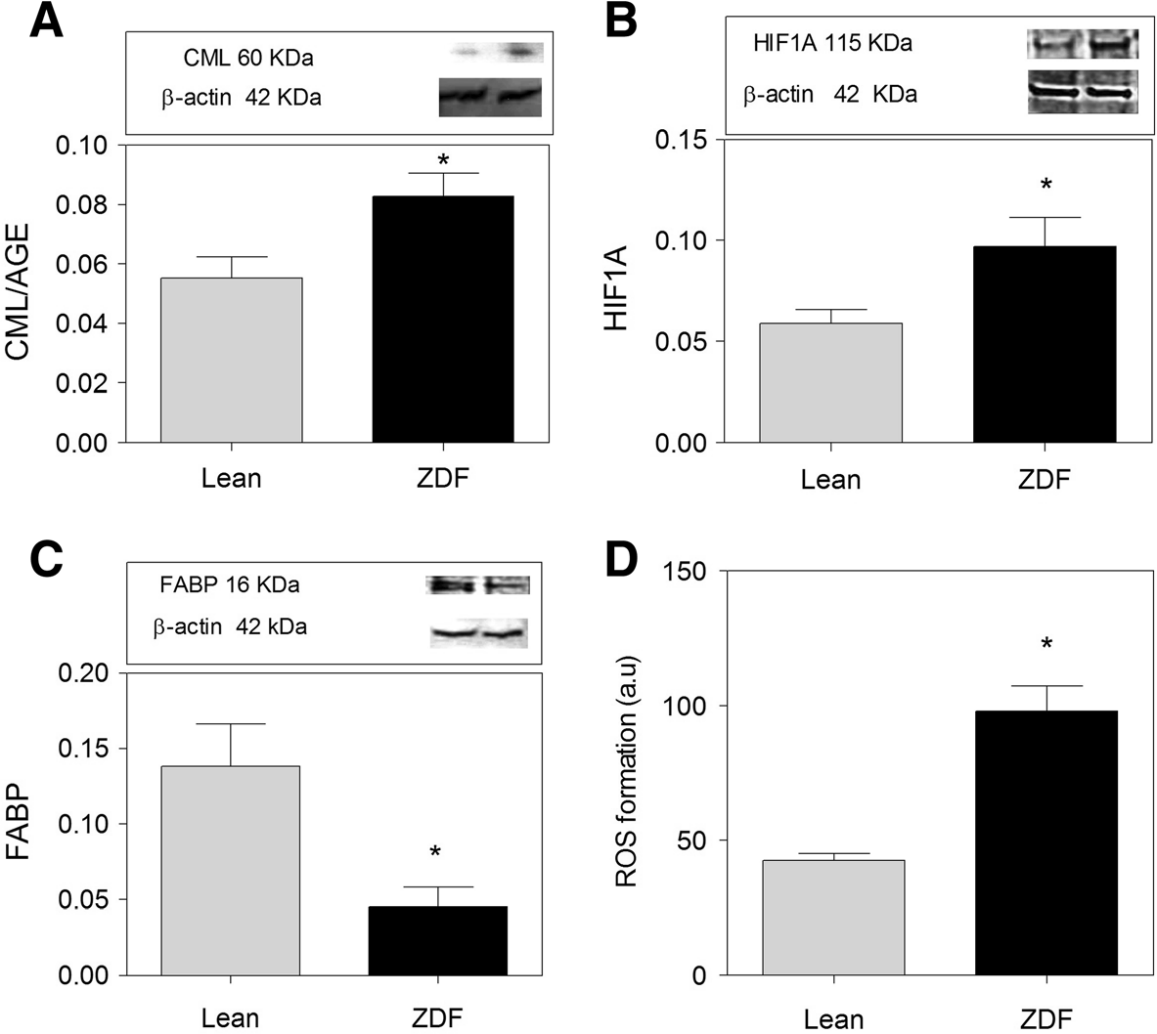
**Oxidative stress and damage in brains of ZDF rats. (A)** Expression of CML/AGE is increased in ZDF compared to Lean. **(B)** HIF1A expression is higher in ZDF brain compared to Lean. **(C)** FABP is 3 times lower in ZDF rats compared to Lean. **D)** ROS formation is higher in ZDF brains compared to Lean. ROS formation is measured by the level of Fluorescin fluorescence. Western blot data showing protein of interest (upper part) and β-actin (lower part). Data are Means ± SEM (n *≥* 5 per group), * <0.0001 (difference to control gray bars), unpaired t-test. Western blot expression is normalized to β-actin; lanes of western blot insets are in the same order as in the X-axis.

Diabetes is associated with increased ROS (Reactive oxygen species) production, part of which is explained by glycosylated protein species inhibiting the electron transport chain of mitochondria [36]. Indeed, ZDF brain showed a slight, but significantly increased level of carboxymethyllysine (CML), indicating the presence of glycosylated protein species (Figure 3A). Moreover, HIF1A (Hypoxia inducible factor 1) levels, known to be upregulated through ROS formation [37], were increased in ZDF brains (Figure 3B). Also, ZDF brain showed a lower expression of FABP (Figure 3C), a protein depleted by ROS damage. Further, a fluorogenic probe was used to assess the formation of ROS in brain tissue. ROS formation in brain homogenate was doubled in ZDF compared to Lean (Figure 3D).

### Brain CBS expression and thiol group content

**Figure 4:**
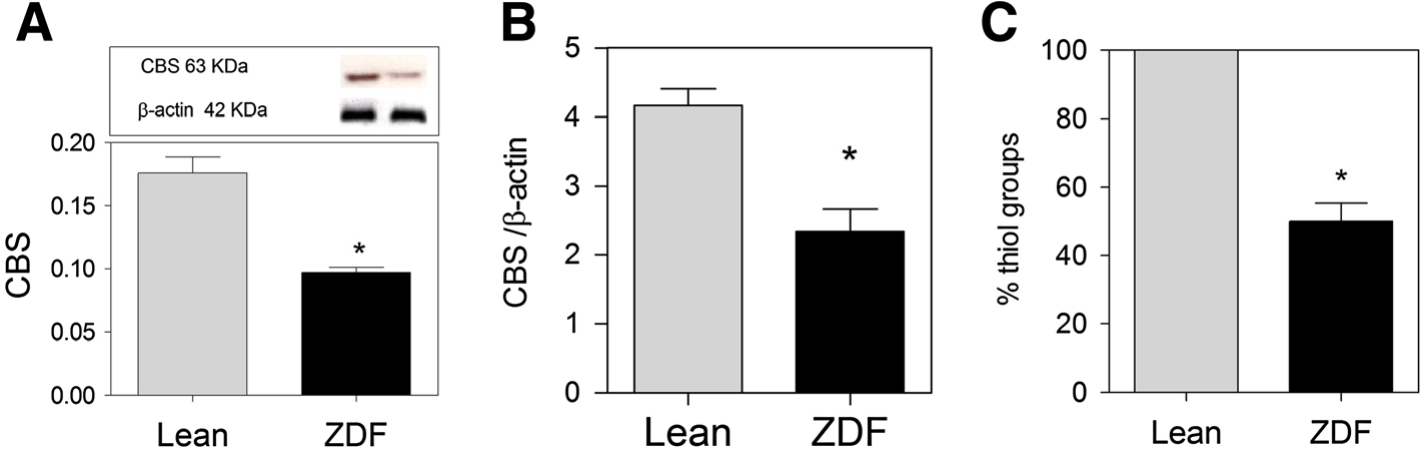
**Thiol groups and CBS (cystathionine beta synthase) expression in ZDF (zucker fatty diabetic rat) and Lean frontal brain. (A)** CBS expression is lower in ZDF brains. **(B)** Real-time PCR analysis of the expression of CBS relative to β-actin, and **(C)** Thiol group level in ZDF is lower than Lean. Western blot data showing protein of interest (upper part) and β-actin (lower part). Data are Means ± SEM (n *≥* 5 per group), * <0.0001 (difference to control gray bars), magnification 40×; unpaired t-test. Western blot expression is normalized to β-actin; lanes of western blot insets are in the same order as in the X-axis.

Expression of CBS (H_2_S producing enzyme) and brain thiol groups were assessed in brain to evaluate the antioxidant defense levels. CBS protein expression (Figure 4A), CBS mRNA levels (Figure 4B) and % thiol groups (Figure 4C) were found to be significantly lower in ZDF brain compared to Lean.

### NaHS treatment diminishes protein aggregation

**Figure 5:**
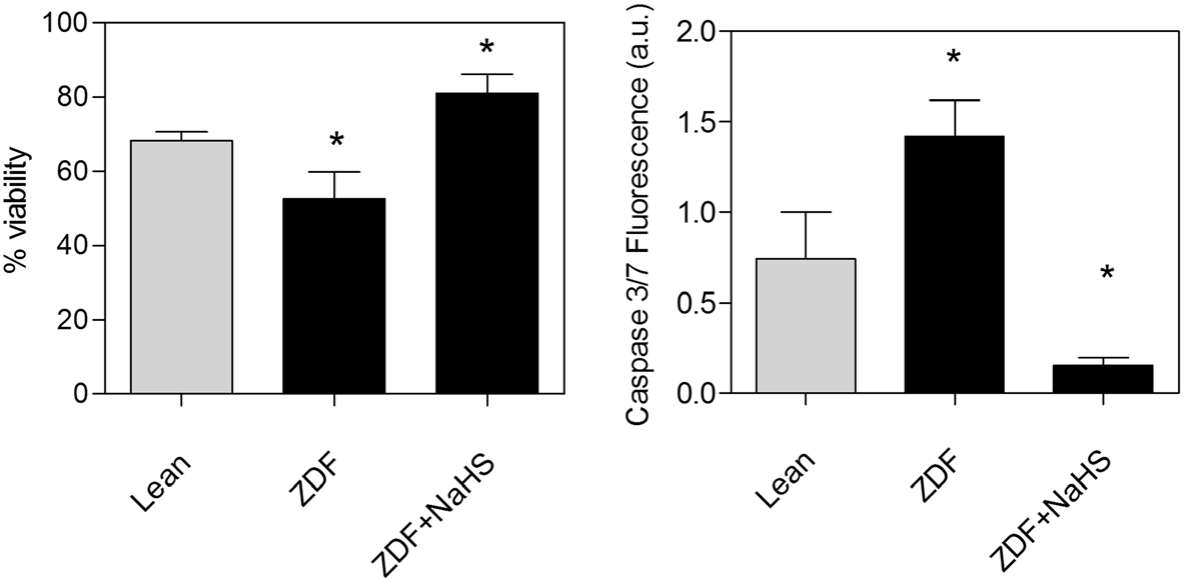
**Tissue viability (MTS assay) and apoptosis (caspase 3/7 activity) in cultured brain slices.** Left panel: Cells dissociated from two days NaHS treated ZDF slices show the highest level of viability with the lowest levels of apoptosis compared to cells in slices from Lean and ZDF or the fully viable non-cultured slices which show 100% viability. Right panel: Cells dissociated from the untreated ZDF slices show the highest caspase activity in culture, which is decreased below the level of Lean by treatment with NaHS. Data are means ± SEM (n *≥* 5 per group), * = different from control gray bars, * < 0.05; One way ANOVA.

**Figure 6:**
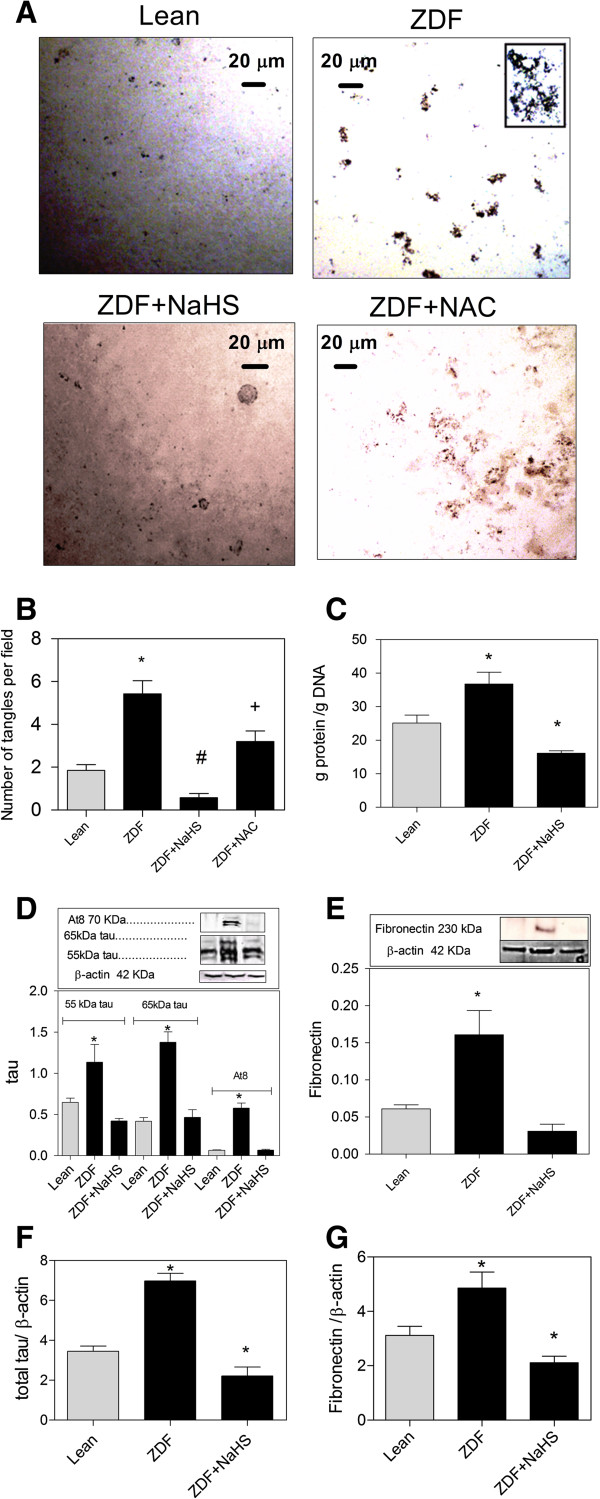
**Treatment with NaHS counteracts protein aggregates in cultured brain slices from ZDF (zucker diabetic fatty rat). (A)** ZDF brain slices retain protein aggregates in culture. Protein aggregates are diminished through NaHS treatment. NAC diminishes protein aggregates in ZDF. **(B)** The doubling of the number of aggregates in ZDF brain slices is lowered to Lean levels by NaHS treatment. NAC lowers protein aggregates in ZDF brain but at a much slower rate. **(C)** NaHS lowers the higher protein synthesis levels in cultured ZDF brains indicated by higher protein/DNA ratio. **(D)** 65 kDa and 55 kDa tau expression is three times higher in ZDF rats compared to Lean and NaHS treatment normalizes tau levels. Also, hyperphosphorylated tau (AT8) is increased in ZDF brains and normalized by NaHS. **(E)** Fibronectin expression is doubled in ZDF rats compared to Lean and is normalized by NaHS treatment. **(F)** Real-time PCR analysis of the expression of total tau relative to β-actin. NaHS lowers total tau expression in ZDF to lower than Lean levels **(G)** Real-time PCR analysis of the expression of fibronectin relative to β-actin. NaHS lowers fibronectin expression in ZDF to lower than Lean levels. Western blot data showing protein of interest (upper part) and β-actin (lower part) from the same samples. Data are means ± SEM (n *≥* 5 per group) *, #, + <0.05 = statistically different, * different from control gray bars in each group, # different from ZDF group, + different from lean, ZDF and ZDF + H_2_S; One way ANOVA. Western blot expression is normalized to β-actin; lanes of western blot insets are in the same order as in the X-axis.

To examine the pharmacological potential of H_2_S in counteracting the defective proteostasis in ZDF brains while excluding the beneficial effects of H_2_S in lowering glucose concentrations in the whole animal [31], H_2_S was administered to cultured brain slices. To this end we used culture medium with 35 mM of glucose, as initial experiments had shown that the normal medium (25 mM glucose) diminishes protein aggregation in brain (data not shown). To this end, slices of ZDF brain were treated with 50 μM NaHS every 10 h for two days. Brain slice viability and apoptosis was substantiated by MTS and caspase 3/7 assays (Figure 5). Cultured brain slices retained protein aggregates (Figure 6A). The number of aggregates was doubled in ZDF brain slices compared to Lean. Treatment of ZDF brain slices with NaHS, however, reduced the number of aggregates to Lean levels (Figure 6B). N-acetyl cysteine (NAC) lowered the number of aggregates but to a lower degree compared to NaHS (Figure 6A-B). Further, protein synthesis level was substantiated by calculating the ratio of protein-to-DNA. This ratio was increased in ZDF brain slices showing higher protein synthesis levels in ZDF, while NaHS counteracted this increase showing inhibitory properties on protein synthesis (Figure 6C). A similar pattern of decrease through NaHS treatment was also found for the other markers. The 65 kDa tau species expression was 3-fold higher in ZDF slices compared to Lean and NaHS treatment was found to normalize tau expression in ZDF brain (Figure 6D). Further, hyperphosphorylated tau, as detected by the AT8 antibody, was present at 70 kDa in ZDF brains which was decreased by NaHS treatment (Figure 6D). Also, fibronectin expression was doubled in ZDF compared to Lean and was normalized by NaHS treatment (Figure 6E).

The levels of total tau mRNA (Figure 6F) and fibronectin mRNA (Figure 6G) was higher in ZDF brains compared to Lean and this increase in mRNA levels was counteracted and lowered to below mRNA levels present in Lean brains.

**Figure 7:**
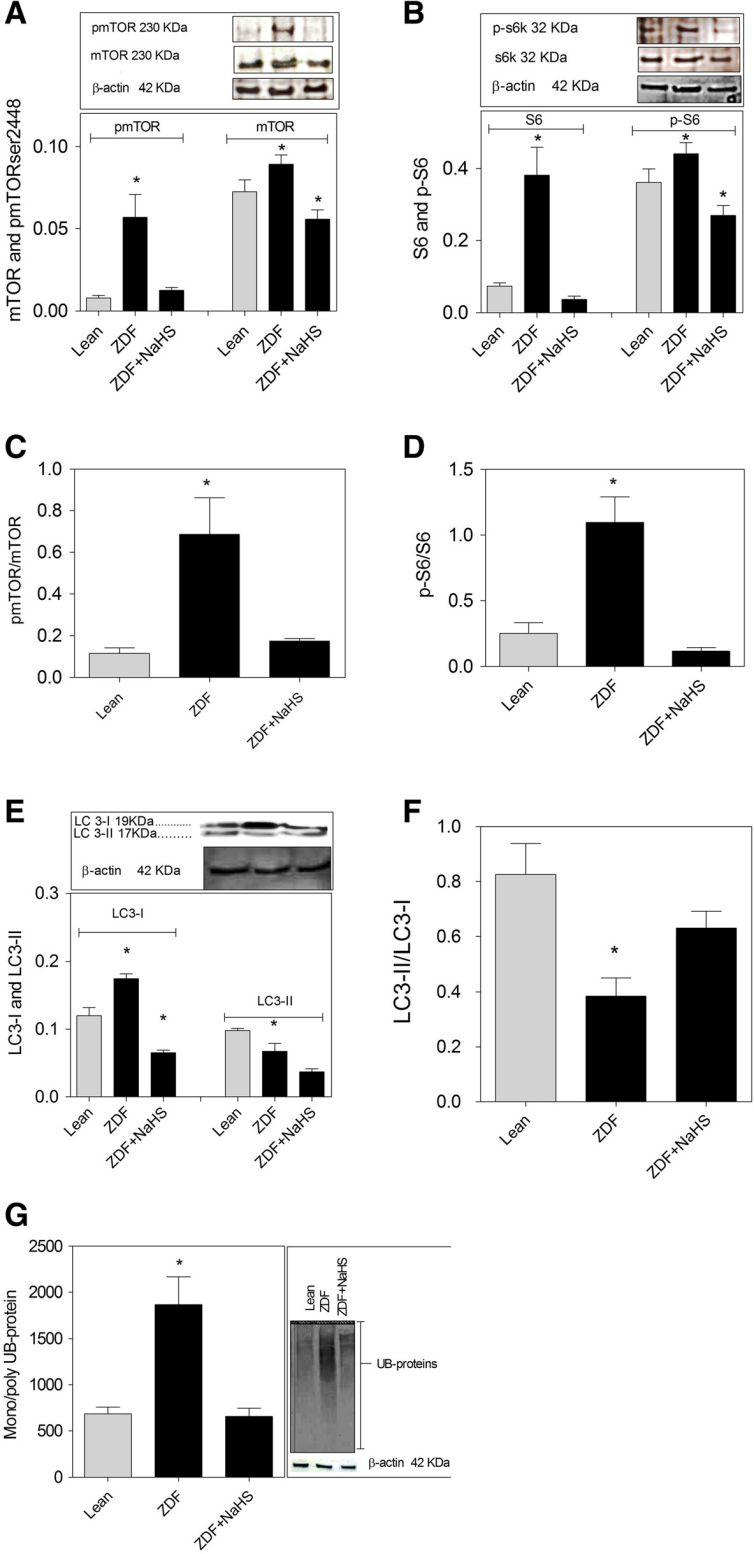
**Treatment with NaHS modulates protein synthesis and degradation pathways in cultured brain slices from ZDF (zucker diabetic fatty rat). (A)** The expression of mTOR and its phosphorylated form (p-mTOR^2448^) is higher in ZDF and is lowered to normal levels by H_2_S treatment. **(B)** The expression of S6 protein and its phosphorylated form (p-S6 ribosomal protein) is higher in ZDF rats and is normalized by NaHS treatment. **(C)** Increased mTOR activity in ZDF is counteracted by NaHS. **(D)** Increased S6 protein activity in ZDF is counteracted by NaHS. **(E)** LC3-I is higher in ZDF brains while LC3-II shows lower expression. NaHS lowers LC3-I expression. **(F)** Autophagy levels are lower in cultured ZDF slices compared to Lean as shown by LC3-II/LC3-I ratio. NaHS increases autophagy levels in ZDF. **(G)** Mono- and polyubiquitinated protein expression is 3 times higher in ZDF brains compared to Lean and is lowered to normal by NaHS. Western blot data showing protein of interest (upper part) and β-actin (lower part) from the same samples. Data are means ± SEM (n *≥* 5 per group), * < 0.05 = different from control gray bars in each group; One way ANOVA. Western blot expression is normalized to β-actin; lanes of western blot insets are in the same order as in the X-axis.

The phosphorylation of mTOR and S6 ribosomal protein was 4 times higher in cultured brain slices from ZDF and both were lowered to normal levels by NaHS treatment (Figure 7A,B). Also the total levels of mTOR (Figure 7A) and S6 ribosomal protein (Figure 7B) were higher in ZDF and were lowered to below the expression observed in Lean brains by NaHS treatment. The ratios of the phosphorylated forms of mTOR and S6 ribosomal protein to the total expression of these proteins in each sample show activation of mTOR (Figure 7C) and S6 ribosomal protein (Figure 7D) in ZDF brains which is fully counteracted by NaHS treatment. LC3-I expression was 2 times higher in ZDF rat brain slices and its level was decreased through NaHS treatment (Figure 7E). The ratio of LC3-II-to-LC3-I in ZDF brains was two times lower compared to Lean brains showing lower autophagy levels in ZDF brains. NaHS strongly increased autophagy levels in ZDF brains (Figure 7F). Mono- and polyubiquinated protein expression was 3 times higher in ZDF brains and was also lowered to Lean levels by NaHS treatment (Figure 7G).

**Figure 8:**
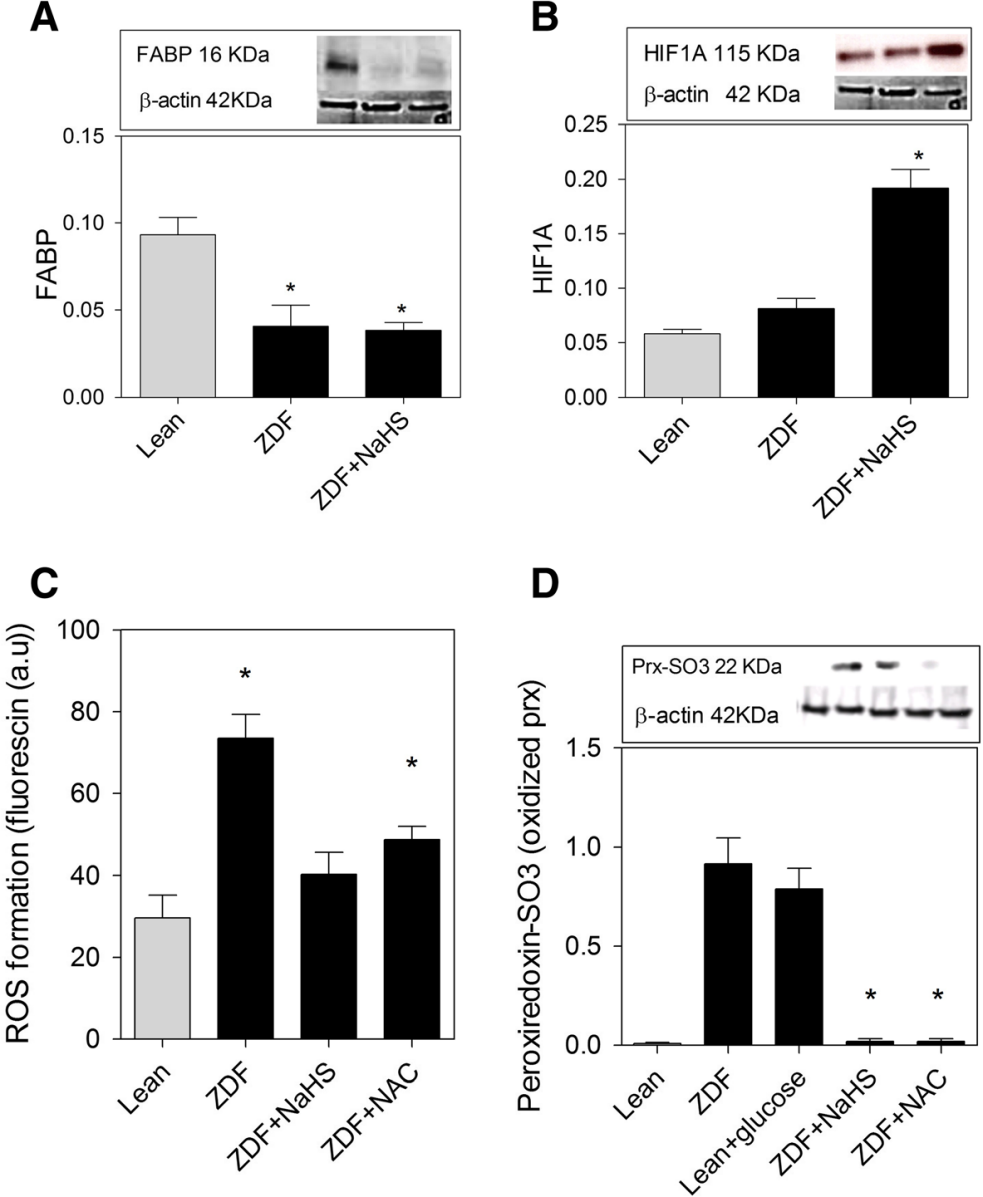
**NaHS counteracts oxidative stress and damage in brain slices of ZDF rats. (A)** HIF1A expression is similar in ZDF and Lean brains but NaHS increases the expression of this protein. **(B)** FABP expression is decreased in ZDF and is unaffected by NaHS treatment. **(C)** ROS production is twice higher in ZDF brains compared to Lean. NaHS an NAC (N-acetyl-L-cysteine) inhibit ROS formation in ZDF slices. ROS formation is measured by the level of Fluorescin fluorescence. **(D)** The oxidized forms of Peroxiredoxins is twice higher in ZDF brain and is reversed to non-oxidized form through NaHS and NAC treatment. Western blot data showing protein of interest (upper part) and β-actin (lower part) from the same samples. Data are means ± SEM (n *≥* 5 per group), * < 0.05 = different from control gray bars; One way ANOVA. Western blot expression is normalized to β-actin; lanes of western blot insets are in the same order as in the X-axis.

HIF1A expression was not found different between ZDF and Lean brains *in vitro*, but NaHS increased the expression of this protein in ZDF (Figure 8A). FABP expression was reduced in ZDF brain slices compared to Lean. NaHS treatment of ZDF brains did not affect FABP expression (Figure 8B). The assessment of ROS levels shows that treatment of Lean at high glucose concentrations did not change ROS levels. ROS production in ZDF brains was inhibited by the addition of NaHS or NAC (Figure 8C).

Peroxiredoxin (Prx) enzyme modulates oxidative stress via its evolutionary conserved cystein (Cys) residues. Under oxidative stress condition, cystein sufinic acid of Prx becomes oxidized. To further study the potential oxidation of Prx enzymes under oxidative stress in ZDF brain, we performed Western blot analysis with oxidized Prx specific antibody, anti-Prx-SO3 in all samples. Our results show that Peroxiredoxins is highly oxidized in ZDF brain and that NaHS and NAC reverse this process (Figure 8D).

Thus, NaHS might be counteracting the defective proteostasis and protein aggregation in cultured ZDF brain slices through reduction in protein synthesis and inhibition of oxidative tress.

## Discussion and conclusion

Our data demonstrate the increase in protein aggregates in brain of ZDF rats probably due to a derailment in protein homeostasis, which was reversed *in vitro* by treatment with NaHS. It appears that several factors might be contributing to protein aggregation in ZDF brains. First, protein synthesis is enhanced, as evidenced by the increase in the expression and activation of mTOR and S6 proteins in ZDF brains. Further, the loss of proper defense against the increased oxidative stress seems to be involved, as demonstrated by decreased levels of FABP, thiol levels and CBS and higher amounts of glycosylated proteins in ZDF. Finally, the clearance mechanisms for deviant proteins seem impaired, as demonstrated by the excess of ubiquitinated proteins (UB-proteins) and a relative impairment of autophagy in ZDF. The exact order and contribution of these factors in ZDF is hard to unravel. Yet our experiments in brain slices cultured under increased glucose conditions suggest that the loss of oxidant defense has an important role, as aggregates from ZDF were abolished by treatment with NaHS, which is known to be a strong cytoprotective antioxidant [27,38]. In addition, we also found NaHS to inhibit the mTOR pathway and the resulting inhibition of protein synthesis is likely contributing to the beneficial effects of NaHS on protein aggregation. Together, our results suggest that diabetes induces protein aggregation in brain of ZDF rats and could potentially provide a basis for possible therapeutic strategies in cognitive disorders related to protein aggregation.

Our data disclose the presence of protein aggregates in ZDF brains. Tau and its hyperphosphorylated form (recognized by AT8 antibody) is one of the main neuronal proteins involved in protein aggregation [39] and has been implicated in the loss of cognitive performance [39]. Hyperphosphorylated tau (p-tau) has been observed previously in the brain of mice models of type 1 and 2 diabetes [40]. Importantly both p-tau [41] and protein aggregates have been found in the brain of streptozotocin (STZ) induced mouse model of type 1 diabetes [42]. Thus, the increased total tau mRNA together with phosphorylation of tau protein in ZDF rat brains, might cause impairment in cellular function and cognitive disorders [43]. Tau hyperphosphorylation in diabetes might be due the tau protein cleavage inducing toxic tau aggregation. Indeed, we observed a dispersed pattern of tau protein staining, which might suggest the cleavage of tau protein in ZDF brain, consistent with a recent report suggesting that tau cleavage is an early event in aggregate pathology [44]. Possibly, high glucose is implicated in ROS formation [45] and the increase in tau cleavage and phosphorylation might be secondary to the chronic presence of ROS in diabetes [46]. The increase in advanced glycation end-products (AGEs) due to protein glycation in ZDF, as exemplified by the increased CML expression, may constitute a core for protein aggregation and probably underlie the process of cognitive decline [47]. High glucose is implicated in the increase in CML in ZDF brains [48] and the increase in fibronectin, a component of extracellular matrix, we observed, may also be due to higher glucose levels and the subsequent excessive ROS formation, as found previously in astrocytes [49].

Our data implicate that several changes in proteostasis might underlie protein aggregate formation in ZDF brains. We found both the expression and phosphorylation of mTOR and S6 ribosomal protein to be higher in ZDF brains. Phosphorylation of these proteins is necessary for protein synthesis. High glucose is known to stimulate mTORC1 to promote protein synthesis [50], which may represent one of the main causes of protein aggregate formation in ZDF brain. Indeed, mTOR inhibitors such as rapamycin protect against aggregate formation in taupathies [51]. In addition to mTOR activation, changes in the two major intracellular protein degradation systems, the autophagy-lysosomal and ubiquitin-proteasome system [52] were observed. Our data suggest a blockade in the autophagy pathway, thus limiting protein turnover, in ZDF brain in view of the absence of transition of LC3-I into LC3-II. Such blockade in autophagy is in line with the results in Alzheimer’s models, in which the induction of autophagy is known to lead to a massive accumulation of autophagy intermediates (vacuoles) with low levels of clearance [53] along dystrophic and degenerating neurites. In addition to its effect on autophagy, proteosomal breakdown of proteins seems also affected, as ZDF had higher expression of ubiquitinated proteins (UB-proteins), which normally are processed by proteosomal degradation [54]. UB-protein aggregates increase in hyperglycemia and endoplasmic reticulum stress [19] and get accumulated in neuronal inclusions [55]. Curiously, UB-proteins accumulated in ZDF brains had a lower molecular weight compared to Lean, which might reflect the increased protein cleavage in ZDF brain. Taken together, in ZDF brains the protein synthesis machinery is activated while protein degradation is impaired, likely becoming the leading cause in the formation of protein aggregates.

ROS damage to proteins exists in brain cells at high glucose levels [45,56] likely representing a second driving force for protein aggregation in diabetes. The presence of increased ROS in ZDF brain is evidenced by the decrease in FABP, and the increases in HIF1A and ROS production. Further, the increase in CML indicates impaired glucose metabolism and formation of oxidized amino acids in ZDF brains [48]. Thus, our results demonstrate that ROS production is likely increased in brain of ZDF rats. Oxidative stress might precede protein deposition [57], but conversely protein aggregates may also increase ROS formation and damage brain cells through oxidization of neuronal components [58] eventually leading to apoptotic or necrotic cell death [5]. Thus, a suitable antioxidant, potentially to some degree inhibits the damage present at high glucose concentrations. Additional loss of oxidant defense also would amplify the detrimental effects of ROS. Amongst all the antioxidants that are available in the body, thiols constitute the major portion of the total body antioxidants and they play a significant role in defense against reactive oxygen species [59]. Here our data show for the first time that the levels of CBS, one of the main H_2_S producing enzymes in brain, and thiol content are significantly lower in ZDF brains.

Treatment with NaHS decreased protein aggregates in cultured slices of ZDF frontal brain. Higher glucose levels (35 mM) in culture medium provided a suitable in vitro model for the experiments, first because according to our previous experiment we found a lower glucose level (25 mM) to diminish the number of protein aggregates in vitro and second because higher glucose levels are known to decrease H_2_S levels [60]. We have previously shown that NaHS modulates mTOR activity and inhibits ROS production in aging Werner syndrome skin fibroblasts while inhibiting protein aggregation in these cells [28]. Moreover, the induction of free thiol groups in an organism might have an important role in delaying the aging process [61]. This research is the first report pointing to the therapeutic potential of H_2_S as NaHS in protecting against protein aggregation in brain. H_2_S may exert its protective effect through various mechanisms. First, H_2_S possesses antioxidant properties and administration of NaHS inhibits reactive oxygen species, and the deposition of ECM (extracellular matrix) components in diabetic rats [62]. Indeed, NaHS treatment upregulated HIF1A in brain slices, potentially protecting cells against oxidative stress while increasing glucose tolerance/metabolism and mediating a neuroprotective response [63]. Its important to note that according to the presented data, having anti-oxidant properties is not enough for a substance to lower protein aggregates in ZDF brain to Lean levels, as NAC which is known to have strong antioxidant activity, inhibited ROS formation but did not show the full beneficial effects of NaHS in lowering protein aggregates. Thus, these results suggest the presence of additional mechanism involved in the beneficial action of NaHS, although ROS inhibition is most probably an important factor. Secondly, H_2_S may act through inhibition of the mTOR pathway. We found H_2_S to inhibit protein synthesis by inhibition of mTOR as also shown previously in kidney cells [50]. A third mechanism of action of H_2_S may constitute of regulation of autophagy. We found H_2_S supplementation to regulate and lower LC3-I expression in ZDF brain. H_2_S treatment led towards higher autophagy as shown by LC3-II/LC3-I ratio. The increase in Sirt-1 levels and Sirt-1 phosphorylation could be another important indicator of higher autophagy levels through H_2_S treatment [28]. As NaHS normalized proteostasis in ZDF brains by inhibiting protein synthesis and gene expression, it would be conceivable that the regulation of autophagy by H_2_S represents a lowered flux of defective proteins to the autophagy pathway. Finally, an effect of H_2_S on cellular metabolism may convey beneficial effects in diabetes. Hyperglycemia is known to inhibit AMP kinase (AMPK) activity leading to cellular damage [64], H_2_S has the potential to preserve mitochondrial function [65] via regulation of mitochondrial ATP-sensitive potassium channel, p38 mitogen-activated protein kinase and c-Jun NH(2)-terminal kinase pathway. Consequently, H_2_S potentially constitutes a suitable treatment against hyperglycemia induced cell damage in case the above pathways are affected [66]. Moreover, H_2_S has been shown to regulate proteostasis in diabetic animals through activation of AMPK [50]. Interestingly, H_2_S also potentiates the function of NMDA-glutamate receptors implicated in long term memory, while protecting proteins against nitration or oxidation [67]. Thus, H_2_S exerts different protective effects which seem to be of importance in the protection of brain tissue from hyperglycemia stress and protein aggregation. The changes in the expression of several proteins probably involved in high glucose induced protein aggregation in ZDF brain and the effect of NaHS on protein aggregation and protein expression in ZDF brain are summarized in Table 2.

**Table 2:** Summary of changes in several important parameters involved in high glucose induced protein aggregation in ZDF brain and the effect of NaHS on them

**Parameters analyzed**	**ZDF brain**	**NaHS + ZDF brain**
ROS levels	↑	↓
Protein aggregates	↑	↓
Protein synthesis (mTOR, S6)	↑	↓
FABP	↓	—
Thiols	↓	↑
CBS	↓	—
CML expression	↑	↓
UB-proteins	↑	↓
Autophagy	↓ (LC3-II↓, LC3-I↑)	↑ (LC3-II↓, LC3-I↓)
Tau and p-tau	↑	↓
Fibronectin	↑	↓

ZDF brain was compared to lean brain as control and NaHS treated ZDF brain was compared to ZDF brain as control. The arrows indicate the changes in each parameter.

Although ZDF animals displayed both an increase in non-fasting blood glucose levels and HbA_1c_, it should be noted that the development of overt diabetes was considerably slower in this batch of ZDF than observed in our previous experiments in the same animal strain [68]. While the reason for this remains elusive, in view of the current data, the animals would be best defined as being pre-diabetic.

This study identified the presence of protein aggregation and its key mechanistic targets in ZDF brain and found it to coincide with lowered brain thiol levels and lowered CBS expression. In addition, we demonstrate for the first time that the administration of H_2_S donor, NaHS, diminishes protein aggregation in the brain. Although we have presented several hypothesis in regards to possible mechanism by which H_2_S inhibits protein aggregation in ZDF brain, our goal here was not to establish a mechanism of action for H_2_S treatment but rather to show that H_2_S inhibits protein aggregates in ZDF brains. This study provides further insight into beneficial effects of H_2_S which may be exploited to protect the diabetic brain.

## Methods

### Animals, glucose measurement and sample preparation

Male Zucker diabetic fatty rats (ZDF, n = 8) and control rats (Lean, n = 8) were obtained from Charles River Laboratories at 6 weeks of age with an average weight of 266 g for ZDF and 203 g for Lean. The animals were fed water and chow ad libitum throughout the experiment till they reached 17 weeks of age. Blood samples were taken every two weeks from week seven to week seventeen. Glucose and HbA_1c_ levels were measured by commercial kits (Roche). At 17 weeks of age, rats were anesthetized by 2.5% isoflurane and sacrificed by exsanguination. Brains were removed and either snap frozen in liquid nitrogen or cut into 200 μm thick slices using a McIlwain tissue chopper and placed in brain nutrition medium/neurobasal medium (Gibco BRL, Cat. No. 21103–049) for *in vitro* experiments. The concentration of glucose in frontal brain homogenates was measured using Accu-Check Aviva (Roche diagnosis, Mannheim, Germany) in which a drop of homogenized brain (n *≥* 5 samples in each group) was placed on the glucose test strip and read by the instrument. All experiments were approved by the Animal Care Committee of the University Medical Center Groningen (Dec 6032B).

### Brain section treatment

To study frontal brain slices in culture, 200 μm thick brain slices were placed in brain nutrition medium/neurobasal medium with 25 mM D-glucose and supplemented with 10% fetal bovine serum and 0.5 mM L-glutamine. As higher glucose levels are suggested to be responsible for the lower baseline levels of H_2_S detected in the medium of cells [60] the above medium was supplemented to a total of 35 mM glucose to create a high state of hyperglycemia, an environment more similar to what is observed *in vivo*. In treated slices, 50 μM NaHS was added to the wells every 10 h until the completion of the experiment at two days of culture. The final concentration of H_2_S in culture medium after the addition of 50 μM NaHS lies in the normal range of brain H_2_S levels found in rats, i.e. between 50 and 160 μM [69]. In all our experiments, NaHS was used at a concentration of 50 μM, as pilot experiments showed this concentration to promote the highest level of cell survival in cultured brain tissue sections according to caspase 3/7 activity assay results, as also previously found by others [69]. Controls did not receive NaHS in the medium. NAC (N-acetyl-L-cysteine) 100 μM, which is the highest achievable concentration in plasma with tolerable oral dosing of NAC, was used to treat brain slices as additional controls to study the effect of a frequently used “antioxidant” in vitro, on ROS levels and protein aggregation in ZDF brains. Brain slices were kept in culture for two days, washed with PBS (phosphate buffered saline) and snap frozen in liquid nitrogen for either tissue staining or Western blot analyses.

### Antibodies and Western blot analysis

Antibodies used were mouse carboxymethyl lysine (R&D system, MAB3247, UK), mouse mono- and polyubiquinilated conjugates (Enzo life Sciences BVBA, BML-PW1210-0025), LC3 (Cell signalling, 2775), B-FABP (Santa Cruz Biotechnology; FL-132; Sc-30088), fibronectin (Santa Cruz, sc-6952), CBS (Santa Cruz SC-46830), phospho-mTOR (Millipore 15–105 Pathway Explorer Anti-phospho-mTOR ^ser 2448^), p-S6 ribosomal protein (S235/236 Cell Signaling D57.2.2E), S6 ribosomal protein (Cell Signaling (5G10) 2217), HIF1A (Santa Cruz, sc-13515), total phosphorylation independent anti-tau (BR134, Cambridge, UK), phosphorylated tau protein (AT8 Ser202/Thr205, Innogenetics, Zwijndrecht, Belgium). Levels of hyperoxidized peroxiredoxins were assessed by Western blotting using rabbit anti-Prx-SO3 antibody (Abcam, ab16830).

Western blot analysis was conducted to investigate protein expression in brain tissue. Frozen frontal brains were homogenized (20% w/v) in ice-cold RIPA lysis buffer (1% Igepal ca-630, 1% SDS, Roche protease and phosphatase inhibitor cocktail in PBS) [70]. The amount of protein was measured by Bradford assay and loading buffer (20 μl) was added to every 50 μg of protein and ran at 100 V for 70 min. Proteins were transferred to nitrocellulose membranes. To use the same membranes to detect different proteins and prevent possible loading interferences in presented data, the membranes were cut at specific places indicated by colored protein ladder, using a sharp surgical scalpel and probed using the appropriate antibody. Protein bands on antibody treated and washed membranes were detected by West Pico Chemiluminescent Substrate (supersignal), photographed by GeneSnap (version 6.07; Syngene, Cambridge, UK) and analyzed with genetool software (version 3.08, SynGene, UK). Protein expression was corrected over β-actin as an internal reference.

### Measurement of protein synthesis in brain tissue

To show the presence of active protein synthesis in ZDF brain and the effect of NaHS treatment on protein synthesis levels, the ratio of protein-to-DNA known as an indice of protein synthesis capacity, was calculated in each sample. Total genomic DNA was isolated from each brain sample using the Nucleospin Tissue Kit (Macherey-Nagel, Duren, Germany). The DNA concentration in each sample was measured using a NanoDrop spectrophotometer (Life Science ND1000, US). Protein concentration in each sample was measured using the Bradford assay.

### Real-time PCR (qPCR)

In addition to Western blot analysis, the expression of fibronectin, tau and CBS in brain slices was analyzed by qPCR. In brief, RNA was extracted from each sample using the Nucleospin tissue kit (Cat NO. 740955.250 Macherey-Nagel, UK). RNA (1 μg) was reversely transcribed in a reaction mixture (20 μl) containing 1 μl of random hexamers, 0.5 μl of RNase inhibitor, deoxynucleotide triphosphates (0.2 μl), 1 μl of reverse transcriptase, 4 μ l RT buffer and Tris buffer (pH 7.4) at 37°C. Specific primers to rat Fibronectin (Forward: AGAGCATACCTCTCAGAG and Reverse: CTGCTCATCAGTTGGGAA), rat total tau (Forward: TGACACGGACGCTGGCCTGAA and Reverse: CACTTGGAGGTCACCTTGCTC), Cystathionine beta synthase (Forward: ATGCTGCAGAAAGGCTTCAT, Reverse: GTGGAAACCAGTCGGTGTCT) and β-actin (Forward: AAGATGACCC AGATCATGTTTGAG and Reverse: ACGTACATGGCTGGGGTGTTG) were synthesized (Base Clear, Netherland). SYBER green qPCR was performed using Bio-rad CFX384 C1000 (USA, CA) and data were quantified by Bio-rad CFX manager 2.0 using reagents from the SYBR Green PCR-Master Mix (Qiagen, Hombrechtikon, Switzerland). The samples in PCR 384 well plate were transferred to the thermal cycler and applied to the following protocol: predenaturation at 94˚C (5 min; 1 cycle) followed by 35 cycles of denaturation at 94˚C (30s), annealing at 57˚C (30 s), extension at 72˚C (45 s) and a final extension at 72˚C (8 min; 1 cycle), followed by a melting curve. All cycle threshold (Ct) values were collected at the exponential phase of the qPCR. All data were normalized to β-actin.

### Histochemistry and microscopy

Flash frozen frontal brains were cut at 15 μm thickness using a cryostat (Leica CM-1800, Chatsworth, CA) at -20°C. Tissue sections were thaw-mounted onto VistaVision HistoBond adhesive slides. The Bielschowsky silver impregnation was performed to reliably stain protein aggregates forming the neurofibrillary tangles [71]. To perform Bielschowsky silver stain, the rehydrated brain sections mounted on slides were covered with 20% AgNO3 and incubated at room temperature in the dark for 20 min. The solution was washed off with distilled water and replaced by ammoniacal silver solution for 20 min in the dark. Then, the solution was tipped off and the slides were immersed in ammonia-water (four drops of ammonia added to 100 ml distilled water). Subsequently, slides were incubated in ammoniacal silver solution with developer (100 ml distilled water, 20 ml formaldehyde, 50 μl concentrated HNO3 and 0.5 g citric acid) to develop the silver stain for 3–6 min. The quality of stain was controlled under the microscope (aggregates were dark brown/black with cluster like conformation). The sections were then immersed in ammonia water for 1 min and then washed twice in distilled water. The reaction was stopped using 5% hypo-sodium thiosulphate for 5 min. Sections were rinsed, dehydrated, cleared and mounted by resinous medium. Aggregates were counted in the frontal brain sections. Ten random fields per slide of brain from four animals in each group were examined.

For immunohistochemistry analysis the mounted sections on slides were subjected to graded ethanol rehydration (100–60%) and brought to water. Hydrogen peroxide activity was blocked for 1 h in peroxidise-blocking buffer (Dako, Denmark, S2023). The sections were then incubated for 1 hr at 37°C with the primary antibodies (1:100 in PBS containing 1% BSA (bovine serum albumin) and 1% rat serum) at room temperature. The slides were washed with PBS, followed by addition of the specific secondary antibodies either fluorescent or non-fluorescent (1:100 in block buffer containing 1% BSA and 1% rat serum) for 1 h at room temperature. Slides were then washed in PBS thrice. Dako mounting medium was incorporated to visualize the nuclei and fix the glass coverslips on fluorescent stains. For non-fluorescent stains AEC (3-Amino-9-Ethylcarbazole) + High sensitivity substrate chromogen (Dako, Denmark k3469) was used to visualize the stain while the nuclei were stained by hematoxylin counter staining. Non-fluorescent stains were then washed in tap water and coverslips were fixed using an aqueous mounting medium (Dako).

### Measurement of reactive oxygen species in brain

Oxygen radicals were determined by a fluorometric method using fluorogenic CM-H2-DCFDA (2,7 dichloroflourescein diacetate). 10 μl of each tissue sample in RIPA was loaded into a 96-well FluoroNunc black Plate. 150 μl of 0.1 M phosphate buffer (pH 7.4) and 10 μl of CM-H2-DCFDA (2,7 dichloroflourescein diacetate) in methanol was added to each well. The plate was incubated in a water bath (37°C) for 30 min and centrifuged at 12,000 × g for 8 min. Fluorescence was measured with a Hitachi fluorescence spectrometer at an excitation wavelength of 488 nm and an emission wavelength of 525 nm. Auto fluorescence was subtracted prior to DCF fluorescence [72].

### Assessment of intracellular thiol groups in brain

Total levels of cellular thiol groups (−SH) in Lean and ZDF brain samples were measured based on the reduction of 5,5 dithiobis-2-nitrobenzoic acid (DTNB, Sigma). Brains were lysed in 10 mM Tris-buffer 1% Triton X-100 (Sigma), followed by centrifugation at 7000 × g for 10 minutes. The supernatant was incubated with phosphate buffer containing DTNB at room temperature for 60 minutes. The quantification was conducted at 412 nm using a microplate reader.

### Statistical analysis

The statistical analyses was based on the analysis of ten slices each from four animals using the One-way ANOVA with Tukey’s test or unpaired student t-test with Welch’s correction. Statistical significance difference was accepted at P < 0.05 (GraphPad Prism version 5).

## Competing interests

The authors declare that they have no competing interests.

## Authors’ contributions

FT, RHH and VMVP designed and conducted analytical experiments, FT, RHH, HB, and VMVP wrote the manuscript, FT, SWL, MHS performed animal experiments and reviewed/edited manuscript. All authors read and approved the final manuscript.
